# Dynamics of oxylipin biosynthesis in systemic inflammation: insights from a large animal model of endotoxemia

**DOI:** 10.3389/fimmu.2025.1595888

**Published:** 2025-06-16

**Authors:** Madison N. Myers, Miguel Chirivi, Jose M. dos Santos Neto, Jair Parales-Girón, Lynn C. Worden, Adam L. Lock, G. Andres Contreras

**Affiliations:** ^1^ Department of Large Animal Clinical Sciences, College of Veterinary Medicine, Michigan State University, East Lansing, MI, United States; ^2^ Department of Animal Science, College of Agriculture and Natural Resources, Michigan State University, East Lansing, MI, United States

**Keywords:** endotoxemia, oxylipins, lipid-based mediators of inflammation, dairy cows, adipose tissue

## Abstract

**Introduction:**

Endotoxemia, marked by the presence of bacterial lipopolysaccharide (LPS) in the bloodstream, induces acute inflammation and is implicated in both mortality and chronic disease across species. LPS stimulates lipolysis and activates cyclooxygenase (COX), lipoxygenase (LOX), and cytochrome P450 (CYP450) enzymes, promoting the synthesis of bioactive lipid mediators known as oxylipins (OXL). However, the dynamics of OXL production during systemic inflammation remain poorly defined, particularly in large animals.

**Methods:**

To investigate OXL responses to endotoxemia, mature Holstein cows were administered intravenous infusions of either LPS or sterile saline (SAL). Plasma samples were collected at baseline (PRE), 2 hours post-infusion (+2H), and 12 hours post-infusion (+12H). OXL profiles were quantified using liquid chromatography-tandem mass spectrometry (LC-MS/MS). Complementary in vitro experiments were conducted using bovine adipocytes exposed to LPS to assess adipocyte-specific OXL release.

**Results:**

LPS-treated cows exhibited classical signs of endotoxemia, including tachycardia, fever, and tachypnea. Plasma OXL profiling revealed significant alterations in arachidonic acid (AA)- and eicosapentaenoic acid (EPA)-derived pathways. Notably, LPS infusion led to persistent increases in COX- and LOX-derived pro-inflammatory OXL, including thromboxane B₂ and hydroxyeicosatetraenoic acids (HETEs), alongside transient elevations in EPA- and docosahexaenoic acid (DHA)-derived pro-resolving mediators. In vitro, LPS stimulation of adipocytes increased the release of AA-based 5-HETE, 6-keto-PGF₁α, linoleic acid (LA)-based 13-HODE, and DHA-based 19,20-DiHDPA.

**Discussion:**

These findings indicate that LPS induces robust activation of pro-inflammatory OXL pathways with limited and transient engagement of pro-resolving lipid mediators. The imbalance may contribute to sustained or dysregulated inflammation. Our study provides novel insights into both systemic and adipocyte-specific OXL dynamics during endotoxemia and highlights their potential as biomarkers and therapeutic targets for modulating inflammation.

## 1 Introduction

Endotoxemia, defined as the presence of bacterial endotoxin in the blood, is a significant contributor to acute death and the progression of chronic diseases in humans and animals. This inflammatory condition results from the translocation of endotoxins, such as lipopolysaccharide (LPS)-containing fragments from the outer membrane of gram-negative bacteria in the gastrointestinal tract, mammary gland, or urogenital tract into systemic circulation following stress or damage to mucosal barriers (1, 2). Interactions between LPS and toll-like receptors (TLR) found on dendritic cells, macrophages, neutrophils, and non-immune cells, such as adipocytes, stimulate the production of reactive oxygen species, pro-inflammatory cytokines, and lipid-based mediators of inflammation (3). In humans, acute exposure to endotoxin on the magnitude of nanograms incites systemic inflammation (4), however, greater concentrations are known to induce septic shock and progressive failure of multiple organ systems (5, 6).

Upon exposure to endotoxins, key enzymatic pathways—cyclooxygenase (COX), cytochrome P450 (CYP), lipoxygenase (LOX)—are activated in immune cells, triggering the oxidation of fatty acids (FA) into the potent class of inflammatory mediators known as oxylipins (OXL) (7). These compounds are broadly classified as either pro- or anti-inflammatory, with omega-6 (n-6)-derived products primarily falling into the former category and those derived from omega-3 (n-3) FA into the latter (8). Importantly, LPS exposure triggers lipolysis, the sequential hydrolysis of triglycerides and release of FA from adipocyte reserves, through its stimulation of toll-like receptor 4 (TLR4) in adipose tissues (AT) (9, 10). The FA released from adipocytes during LPS-induced lipolysis may subsequently serve as substrates for the biosynthesis of OXL, which could amplify or attenuate the inflammatory cascade initiated by endotoxemia (11, 12).

OXL exhibit diverse biological effects dependent upon their FA precursors and biosynthetic pathways. For example, the n-6 FA arachidonic acid (C20:4, n-6; AA) and linoleic acid (C18:2, n-6; LA) give rise to predominantly pro-inflammatory OXL, including leukotrienes (LT), prostanoids, and hydroxylated metabolites such as hydroxyeicosatetraenoic acids (HETE) and hydroxyoctadecaenoic acids (HODE), respectively, which stimulate neutrophil recruitment, vascular permeability, and pain sensitization (13–15). In contrast, n-3 FA-based OXL, such as resolvins (Rv), protectins (PD), and maresins (MaR) from docosahexaenoic acid (C22:6, n-3; DHA), docosapentaenoic acid (C22:5, n-3; DPA), and eicosapentaenoic acid (C20:5, n-3; EPA), act as specialized pro-resolving mediators (SPM), which promote inflammatory resolution and tissue repair by limiting immune cell infiltration and activating anti-inflammatory pathways (16, 17). Together, the balance of pro- and anti-inflammatory OXL plays a critical role in shaping immune responses during inflammation, with their dysregulation implicated in chronic diseases and metabolic disorders (18). Therefore, OXL represent a critical link between lipid metabolism and inflammation, with their synthesis tightly regulated by enzymatic pathways and the availability of FA substrates. Given their dual roles as inflammatory mediators and potential biomarkers, understanding OXL dynamics is essential for deciphering the complex interplay between systemic inflammation and metabolic health.

Considering the capacity of OXL to alter inflammatory responses, understanding their biology is crucial in the context of clinical endotoxemia, which affects both human and animal species. This condition contributes to significant morbidity and mortality rates and sizable financial burdens tied to treatment costs, preventative measures, and management of secondary illnesses (19–22). Of note, dairy cattle are particularly susceptible to endotoxemia due to several physiological and anatomical factors that facilitate the translocation of LPS into systemic circulation: these include changes to the diet, the unique anatomy of the ruminant gastrointestinal tract, and high incidence of reproductive tract injuries that occur during calving (23). Together, these factors create a physiological environment conducive to LPS translocation and make dairy cattle an ideal model to study the intricate relationships between endotoxemia, lipolysis, and OXL biosynthesis. However, despite extensive research on endotoxemia in monogastric species, whether a bovine model can accurately mimic the effects of acute LPS exposure observed in humans and rodents remains unknown (24). Additionally, the timeline from systemic LPS exposure to changes in plasma OXL profiles is, to the authors’ knowledge, unestablished. Furthermore, the involvement of specific enzymatic pathways that regulate OXL biosynthesis remains unidentified and unexplored in the context of endotoxemia, particularly as they pertain to AT. Therefore, identifying systemic and adipocyte-specific alterations in OXL levels and the pathways behind their production may provide evidence for their use as biomarkers, particularly in acute infection, and aid in our understanding the onset and progression of chronic diseases associated with LPS exposure.

Therefore, the objective of this study was to establish these dynamics using an *in vivo* bovine model of endotoxemia, providing translational insights relevant to human and animal health. Using bovine *in vivo* and *in vitro* models of endotoxemia, we define the dynamics of OXL profiles during endotoxin challenges. Our results demonstrate temporal changes in the biosynthesis of OXL derived from n-3 and n-6 FA that characterize, in part, the progression of the inflammatory response during endotoxemia.

## 2 Materials and methods

### 2.1 Animals

All animal use procedures were approved by the Institutional Animal Care and Use Committee (IACUC #201900248) at Michigan State University (East Lansing, Michigan) and were performed in accordance with local and federal guidelines.

Eight multiparous (2.5 ± 1.06; mean parity ± SD) lactating Holstein dairy cows from the Michigan State University Dairy Cattle Teaching and Research Center were enrolled for the *in vivo* part of this study. At enrollment, cows averaged (mean ± SD), 204 ± 22 d in milk and, on the scale from 1 to 5 as defined by Wildman et al., a body condition score of 3.26 ± 0.15 (25). All cows received a common diet formulated to meet the nutrient requirements of the average lactating cow (26). Cows were housed in individual tie stalls bedded with sawdust, cleaned twice daily, and equipped with automatic waterers. Cows were fed 115% expected intake at 7000 h daily. Feed access was blocked from 0500 to 0700 h for orts (uneaten feed) collection and measurements along with offering of new feed. Cows were milked three times per day at 0400, 1200 and 2000 h. Standard herd health examinations were conducted throughout the study.

### 2.2 *In vivo* model of endotoxemia

#### 2.2.1 Bovine model of endotoxemia

The study took place over a 12-d period. For the first 5 d, cows were monitored, and baseline clinical data was collected. On d 5, an indwelling jugular catheter was inserted as described previously (27). Briefly, each cow was restrained, and the area over the jugular vein was clipped and prepared with a surgical scrub. Following, local anesthetic was injected subcutaneously into the area superficial to and immediately surrounding the insertion site (40mL lidocaine hydrochloride 2% formulation, VetOne, Paris, France). After, scrubbing was repeated. An indwelling catheter was then placed and secured by non-resorbable suturing of tape wings around the catheter’s base to the skin.

Bacterial lipopolysaccharide (LPS; derived from *E. coli* O55:B5; Cat #L6529, Sigma-Aldrich, St. Louis, MO) was dissolved in pyrogen-free distilled water at a concentration of 0.1 mg/mL, filtered (0.22 µm), and stored in a sterile glass bottle at 4°C. On d 6, the LPS solution was diluted in 100 mL of sterile 0.9% sodium chloride solution (SAL) according to each cow’s bodyweight (BW; determined at the start of the study using an electronic scale) such that each cow received 1 µg LPS/kg BW (n=4) or 100 mL of SAL only (n=4), which served as negative control. The dosage and serotype of LPS were selected based on prior research conducted by our group (28). Treatments were transported to the farm at 37°C. Catheters were flushed with heparinized saline prior to treatments (SAL, LPS) being intravenously infused over a 20 min period. Infusions took place prior to morning feeding. All animals were monitored closely, and none exhibited clinical symptoms (e.g., excessive fever or shock) which would otherwise require removal from the study and veterinary medical intervention.

#### 2.2.2 *In vivo* sample and data collection

Throughout the experiment, heart and respiratory rate, rectal temperature, and clinical symptoms were monitored and recorded 2 h prior to SAL and LPS infusions (PRE), immediately after (0H) and then hourly for the first 6 h following infusions, and then at 12 h post-infusions (+12H). Heart rate was determined by stethoscope. Rectal temperature was measured using a digital thermometer. Respiratory rate was determined by counting flank movements for a 30 s interval and multiplying by 2 to convert to breaths per min. Heart rates above 84 beats per minute were considered tachycardic (29). Rectal temperatures exceeding 39.3°C were considered febrile (30). Respiratory rates exceeding 36 breaths per minute were considered tachypneic (31).

Blood was drawn via coccygeal venipuncture using coated collection tubes (heparinized saline). Blood samples were collected immediately before infusions (PRE), 2 h after infusion (+2H), and 12 h after infusion (+12H) and centrifuged at 2,000 × *g* for 10 min at 4°C for collection of plasma, which was flash frozen and stored at −80°C until further analysis.

Feed intake and milk production were monitored throughout the study and are reported in the companion paper (32).

### 2.3 *In vitro* model of endotoxemia

#### 2.3.1 Adipose tissue sample collection

Six healthy, non-lactating, non-gestating, multiparous Holstein dairy cows were selected from a local abattoir as previously described by our group (33). Briefly, animals were assigned a body condition score (BCS) by a trained technician prior to slaughter by captive bolt and jugular exsanguination. Only cows with a BCS between 3.25 and 3.75 and no internal signs of disease upon postmortem inspection were included in the study. From the right paralumbar fossa (flank) region, 5 g of subcutaneous AT were collected, immediately placed in filtered Krebs-ringer bicarbonate buffer (KRBB, pH 7.4), and transported to the laboratory at 37°C.

#### 2.3.2 Adipocyte Progenitor Isolation and Exposure of Adipocytes to Endotoxin

From the AT, pre-adipocytes were isolated by outgrowth adhesion, expanded, and matured into adipocytes for 7 d as outlined in (33). Following, mature adipocytes were incubated for 3 h in serum-free culture medium consisting of KRBB+2% bovine serum albumin only (BAS) or in media containing 1 µg/mL lipopolysaccharide (LPS) as reported in the companion paper (32). Then, culture medium was collected, snap-frozen, and stored at –80°C until further analysis. Protein content was quantified within each culture well using the Pierce BCA Protein Assay Kit (Thermo Scientific; Waltham, MA) and used to normalize OXL concentrations in the culture medium to cell abundances in each well (28).

### 2.4 Targeted lipidomic analyses

Solid phase extractions and quantitative targeted lipidomic analyses of plasma and adipocyte culture medium samples were performed by the Wayne State University Lipidomics Core (Detroit, Michigan) using high performance liquid chromatography-tandem mass spectrometry (HPLC-MS/MS). For recovery and quantitation, samples (1 mL plasma or culture medium) were spiked and mixed thoroughly with 1 ng of each of the following internal standards: 15(S)-HETE-d_8_, leukotriene B_4_-d_4_, resolvin D2-d_5_, 14 (15)-EpETrE-d_11_, and prostaglandin E_1_-d_4_. Polyunsaturated fatty acids and their metabolites were then extracted using C18 extraction columns as described previously (34–37). In brief, spiked samples were applied to conditioned C18 cartridges, washed with HPLC-grade water, hexane, and dried under vacuum. Following, 0.5 mL methanol was used to elute samples from cartridges. The eluate was then dried under nitrogen, and the residue redissolved in methanol:25 mM aqueous ammonium acetate (1:1).

Following, HPLC was performed using Luna C18 (3µm, 2.1x150 mm, StrataX SPE cartridge, 30 mg sorbent; Phenomenex, Torrance, CA) columns. The mobile phase consisted of a gradient between methanol-water-acetonitrile at 10:85:5 v/v and 90:5:5 v/v, both with 0.1% ammonium acetate. The gradient program consisted of 0–1 min, 50%; 1–8 min, 50-80%; 8–15 min, 80-95%; and 15–17 min, 95%. The flow rate was 0.2 mL/min. The HPLC eluate was then directly introduced to the ESI source of the QTRAP7500 triple quadrupole mass analyzer (SCIEX, Framingham, MA) operated in negative ion mode. The operating conditions were as follows: Curtain gas and GS1 at 40 psi, GS2 at 70 psi, temperature at 500˚C, ion spray voltage at -2500 V, collision gas at 12 psi, declustering potential at -60 V, and entrance potential at -7 V. The eluate was monitored by Multiple Reaction Monitoring (MRM) to detect unique molecular ion-daughter ion combinations for each transition to monitor a total of 6 lipids and 83 lipid mediators. The MRM monitored each transition for 120 sec around the established retention time for each lipid mediator. Optimixed collisional energies (18–35 eV) and collision cell exit potentials (7–10 V) are used for each MRM transition. Mass spectra for each detected lipid mediator were recorded using the Enhanced Product Ion (EPI) feature to verify identities of detected peaks and MRM transition and retention times matched the standards. Data were collected and MRM transition chromatograms were quantified using SCIEX OS 3.4 Software (SCIEX). In each chromatogram, internal standard signals were used for recovery normalization and quantitation of each lipid mediator. Details on MRM-internal standard mapping are included in **Supplementary File S1**.

### 2.5 Statistical analysis

To ensure robustness and accuracy in our evaluation of treatment effects, OXL with insufficient or inconsistent detection across samples (<3 observations per group per time relative to infusion) were excluded from the statistical analysis. Health parameters and plasma OXL data were analyzed using the GLIMMIX procedure in SAS (SAS On Demand for Academics, SAS Institute, Cary, NC). For *in vivo* data, variables evaluated over time were analyzed with repeated measures using the random effect of animal and the fixed effects of treatment, time, and their interactions. PRE OXL concentration values were included as covariates. For *in vitro* data, the fixed effect of treatment was assessed. The normality of all variables was verified using the Shapiro-Wilk Test (*P*<0.05). Residuals of each model were checked and found to be normally distributed. Results are presented as mean ± SEM unless otherwise stated. Significance was declared at *P ≤* 0.05 and tendencies at *P ≤* 0.10.

The relative impacts of *in vivo* LPS infusion on 13 user-defined OXL biosynthetic pathways were assessed by quantitative enrichment analysis on the web-based platform MetaboAnalyst 6.0 (38) as approached in (39). This method employs the global test algorithm and uses a generalized linear model to produce a Q-statistic for each self-defined set of metabolites. Of the 75 metabolites detected, 69 were recognized across the software’s referenced databases and were included in our analyses. Pathways were considered significantly enriched with Holm *P*<0.00625 (=0.05/8) and false discovery rate (FDR) <5%. Principal component analyses were performed using the MetaboAnalyst 6.0 platform to determine if exposure to endotoxin (e.g., LPS versus SAL) and timing relevant to exposure (e.g., +2H, +12H) resulted in distinct plasma OXL profiles. These results, which facilitate the visualization of sample clustering across treatment and time groups, are presented as score plots.

Correlation analyses were conducted in JMP Pro (v.17, SAS Institute Inc.). To initially screen OXL for associations with physiological parameters, the *Fit Y by X* function was used to evaluate all OXL in relation to respiratory rate, heart rate, and rectal temperature across treatments. The top ten OXL exhibiting linear correlations were selected based on the strength (correlation coefficient) and statistical confidence (*P*-value) of each correlation. Top-ranking OXL were then analyzed alongside all physiological parameters and OXL using the *Multivariate and Correlations* function, allowing for a comprehensive assessment of correlations across health parameters and OXL profiles.

## 3 Results

### 3.1 Intravenous LPS infusion induces clinical signs of endotoxemia in dairy cows

**Figure 1:**
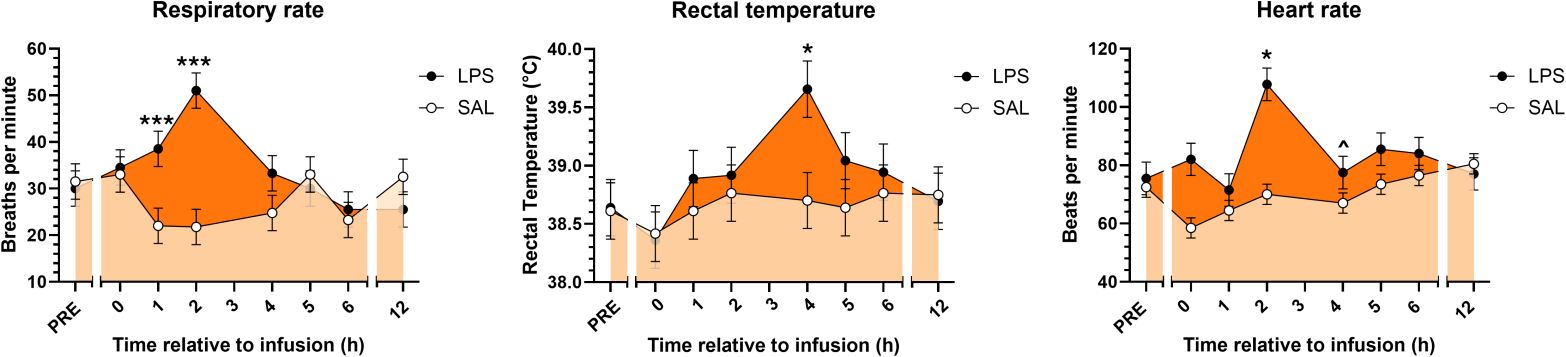
Respiratory rates, rectal temperatures, and heart rates of cows prior to and following continuous intravenous infusion with saline (SAL; open circles; light orange shading) or lipopolysaccharide (LPS, 1 µg LPS/kg body weight; closed circles; dark orange shading) over a 20-min period. Symbols above points indicate differences (*P*<0.001***, <0.05*) and tendencies for differences (*P*<0.10^) between treatments at each time point.

*In vivo* infusion of LPS induced signs and symptoms characteristic of endotoxemia. Following LPS infusion, cows exhibited tachypnea, fever, and tachycardia, whereas animals that received SAL did not (**Figure 1**). Exposure to LPS increased respiratory rate immediately following infusion through +2H compared to SAL and gradually returned to normal (eupneic) levels by 5 h post-infusion (**Figure 1**). LPS induced fevers in cows, with rectal temperatures peaking at approximately 4 h post-infusion, and temperatures transiently returning to normal (afebrile) temperatures by +12H (**Figure 1**). As expected, LPS exposure induced excessive salivation, increased urination and defecation, and coldness of the extremities, particularly of the ears (data not shown). As reported in the companion paper, LPS reduced milk yield and feed intake during the first 3 d following LPS infusion (32).

### 3.2 Endotoxemia enhances arachidonic acid- and eicosapentaenoic acid-based oxylipins derived from COX, LOX, and CYP pathways

**Figure 2:**
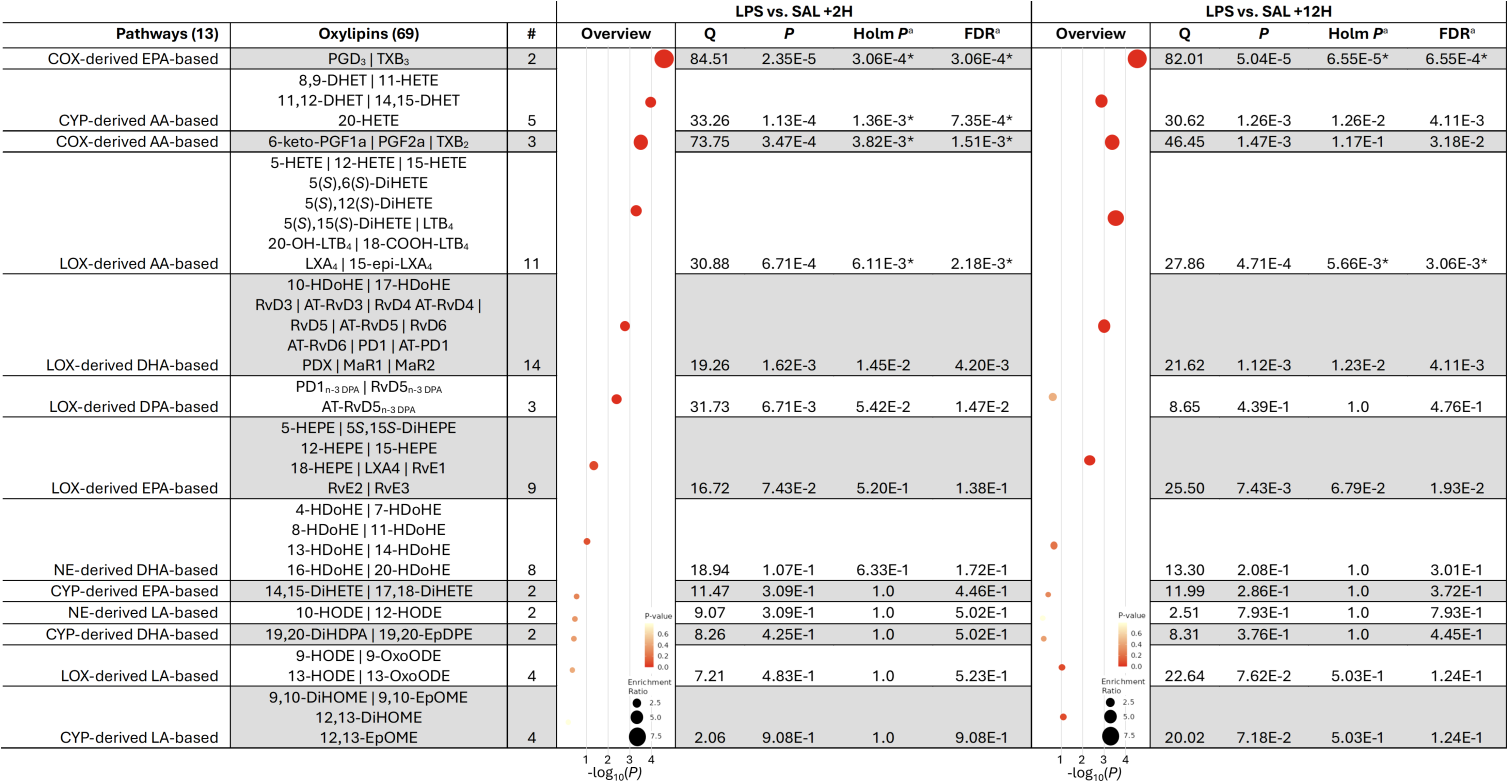
Metabolite set enrichment analysis of oxylipin concentrations in the plasma of dairy cows infused with bacterial lipopolysaccharide (LPS) relative to those given saline (SAL) at 2 (+2H) and 12 h (+12H) post-infusion. Pathways were considered significantly enriched when the ^a^Holm *P*<6.25E-3 and ^a^FDR ^<^5% (denoted by *). AA, Arachidonic acid; COX, cyclooxygenase; CYP, cytochrome P450 epoxygenase; EPA, eicosapentaenoic acid; DHA, docosahexaenoic acid; LOX, lipoxygenase; NE, non-enzymatic.

Our initial analysis assessed the effect of systemic endotoxin exposure on plasma eicosanoid profile. Of the 13 pathways and corresponding metabolite sets analyzed, at +2H, LPS altered COX-derived EPA-based, CYP-derived AA-based, COX-derived AA-based, and LOX-derived AA pathways compared to SAL (**Figure 2**). Elevations in the COX-derived EPA-based and LOX-derived AA-based OXL metabolite sets were sustained between LPS and SAL through +12H (**Figure 2**).

**Figure 3:**
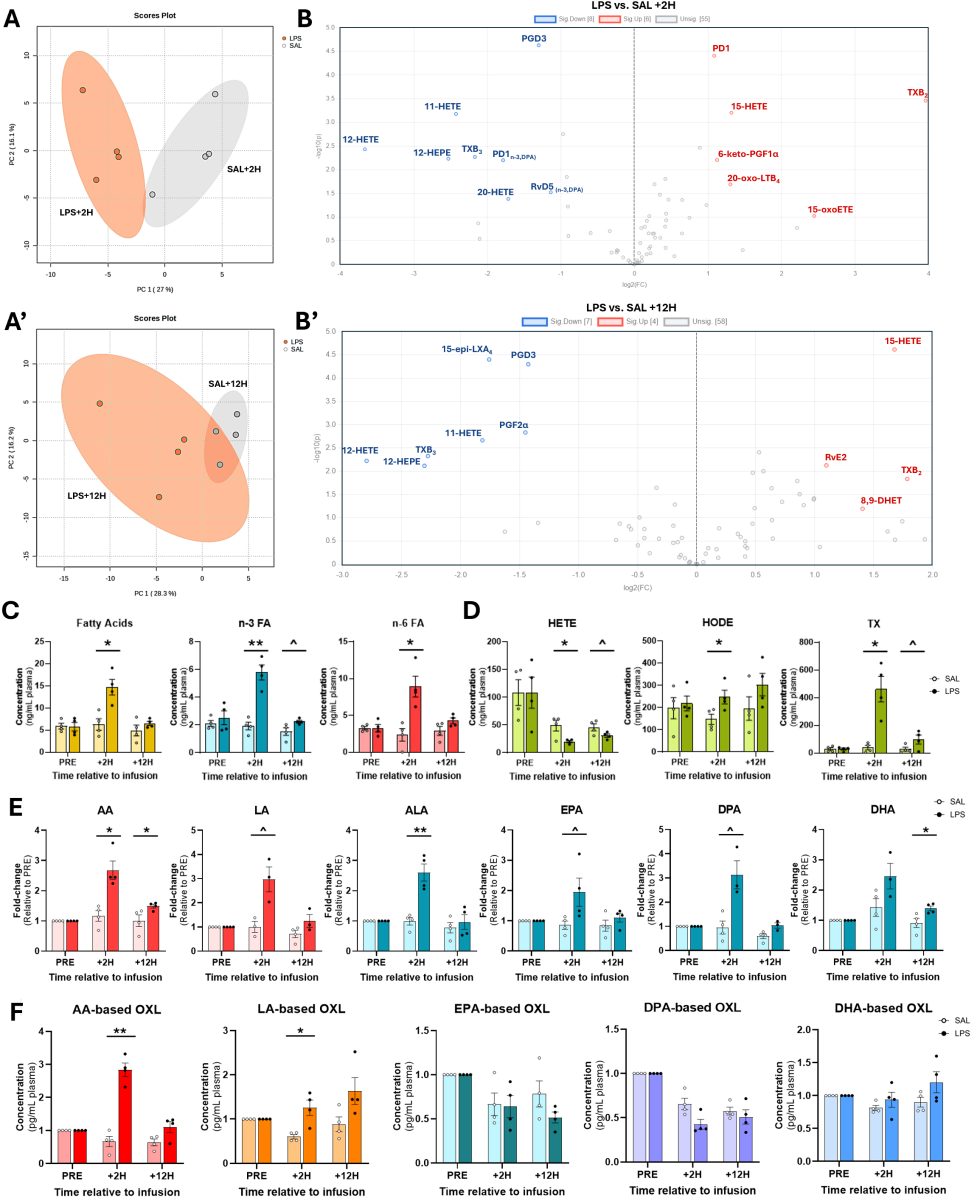
Plasma oxylipin and fatty acid profiles are modulated by endotoxin exposure. **(A)** Principal component analysis plots demonstrate distinct separation of eicosanoid profiles between bacterial endotoxin (lipopolysaccharide, LPS)- and saline (SAL)-treated cows at 2 (+2H) and 12 hours (+12H) after infusion. **(B)** Volcano plots depict up- (red) and down-regulated (blue) oxylipin (OXL) concentrations in LPS relative to SAL. Plots were created in MetaboAnalyst using OXL fold-changes and corresponding *P*-values. **(C)** Bar plots depicting relative plasma concentrations (ng/mL) of summed fatty acids (FA; n-3 and n-6 combined; shown in gold bars), n-3 FA (alpha-linolenic, docosahexaenoic, docosapentaenoic, and eicosapentaenoic acids; shown in blue bars), n-6 FA (arachidonic and linoleic acids; shown in red bars), and **(D)** hydroxyeicosatetraenoic acids (HETE; 5-, 11-, 12-, 15-, and 20-HETE), hydroxy-octadecadienoic acids (HODE; 9-, 10-, 12-, and 13-HODE), and thromboxanes (TX; TXB_2_ and TXB_3_). **(E)** Relative abundance of arachidonic (C20:4, n-6; AA), α-linolenic (C18:3, n-3; ALA), docosahexaenoic (C22:6, n-3; DHA), docosapentaenoic (C22:5, n-3; DPA), eicosapentaenoic (C20:5, n-3; EPA), and linoleic (C18:2, n-6; LA) acids prior to (PRE), 2 h after (+2H), and 12 h after (+12H) infusions with saline (SAL; open circles) and lipopolysaccharide (LPS; closed circles). OXL concentrations are displayed as fold-changes relative to PRE levels for each treatment group. Red bars represent n-6 FA, and blue, n-3 FA. **(F)** Concentrations (in pg/mL plasma) of fatty acid-derived OXL in cows’ plasma. In **(C–F)**, symbols above bars indicate differences (*P*<0.01**, <0.05*) and tendencies (*P*<0.10^) between treatment groups and dots represent individual datapoints.

Principal component analyses (PCA) revealed distinct separation of OXL profiles between LPS- and SAL treatments at +2H (**Figure 3A**); however, plasma OXL profiles trended toward convergence in LPS and SAL at +12H (**Figure 3A’**). Of the 75 quantified, LPS significantly upregulated plasma levels of 6 and downregulated 8 OXL at +2H compared to SAL (**Figure 3B**). At +12H, however, LPS upregulated abundances of 4 and downregulated 7 OXL relative to SAL (**Figure 3B’**). Compared to SAL, LPS increased plasma content of total FA, total n-3 FA, total n-6 FA, and OXL by species relative to SAL (**Figure 3C**). Compared to SAL, LPS increased plasma content of total FA, n-3, n-6, HODE, and TX at +2H (**Figures 3C, D**). Interestingly, elevations in n-3 FA and TX tended to be sustained in LPS through +12H (**Figures 3C, D**). In contrast, LPS reduced total HETE relative to SAL at +2H, with a similar trend observed at +12H (**Figure 3D**).

### 3.3 Endotoxin exposure increases plasma n-3 and n-6 fatty acids and oxylipin derivatives

Compared to SAL, LPS increased plasma AA (2.6-fold) and ALA (2.5-fold) at +2H (**Figure 3E**). Additionally, LPS tended to increase DPA, EPA, and LA at +2H by 3-, 1.9-, and 3-fold, respectively, relative to SAL. At +12H, however, LPS increased AA and DHA 1.4- and 1.5-fold, respectively, over SAL (**Figure 3E**). Corresponding with elevations in relative precursor FA, LPS increased total AA-derived and LA-derived OXL levels in plasma at +2H compared to SAL (**Figure 3F**). There was no effect of treatment observed on plasma EPA-, DPA-, and DHA-derived OXL levels (**Figure 3F**).

### 3.4 Arachidonic acid-derived oxylipin levels are differentially modulated during endotoxemia

**Figure 4:**
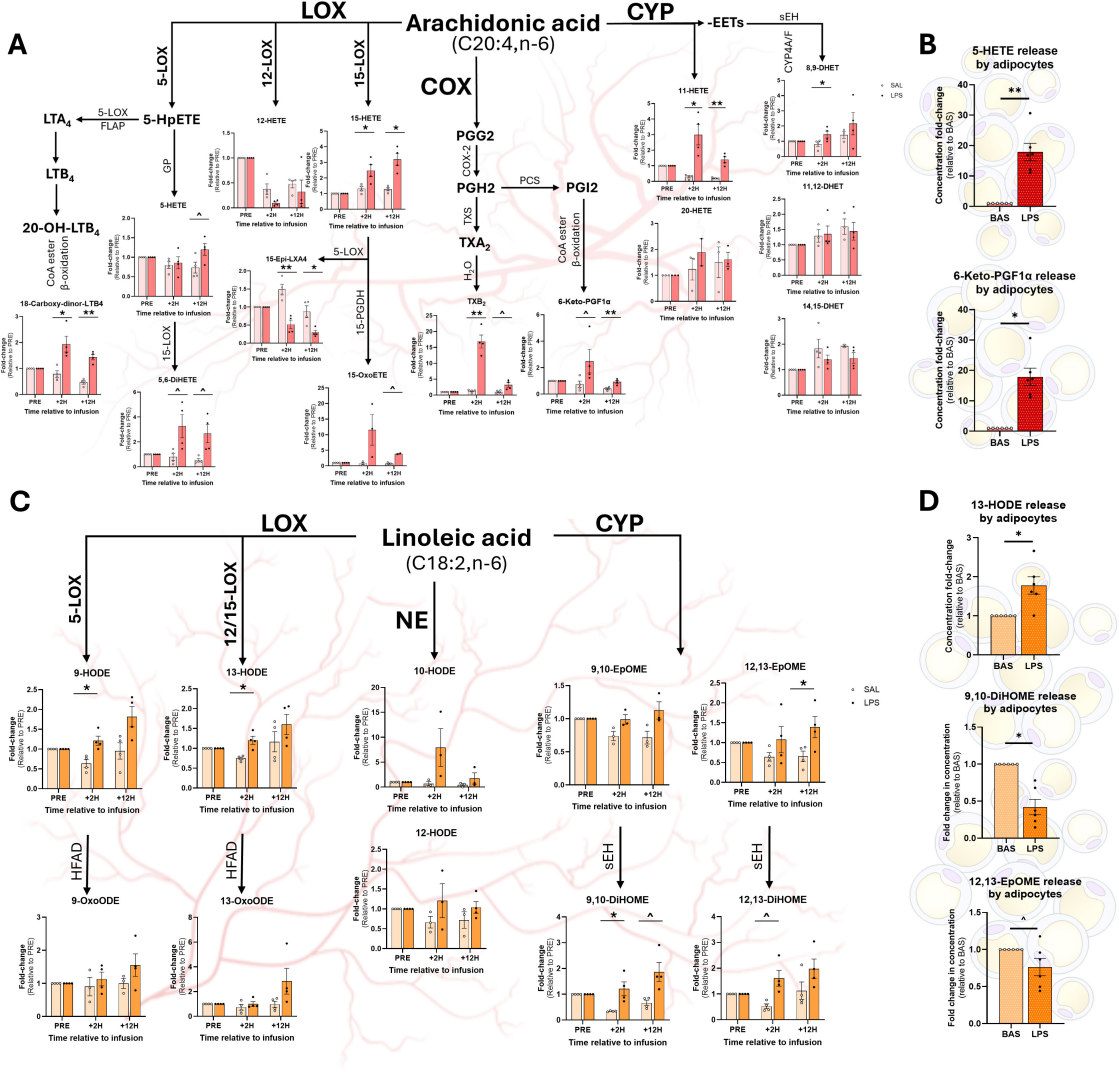
Relative abundance of omega-6 fatty acid-based oxylipins (OXL) in plasma and adipocyte culture medium following treatment with LPS. Arachidonic acid (AA; C20:4, n-6)-derived OXL **(A, B)** and linoleic acid (LA; C18:2, n-6)-derived OXL **(C, D)** in cows’ plasma and adipocyte medium, respectively. OXL were quantified in cows’ plasma before (PRE) and after (2 hours, +2H; 12 hours, +12H) saline (SAL; open circles) and endotoxin (LPS; closed circles) infusion (Panels A and C). Pathways of origin are shown for each oxylipin (OXL) and include enzymatic 5-, 12-, and 15-lipoxygenases (5-LOX, 12-LOX, 15-LOX), cyclooxygenase (COX), cytochrome P450 (CYP), and non-enzymatic (NE) synthesis. Additional reactions are performed by the 5-LOX-activating protein (FLAP), fatty acyl CoA esters (CoA ester) and beta-oxidation (β-oxidation), glutathione peroxidase (GP), 15-prostaglandin dehydrogenase (15-PGDH), prostacyclin synthase (PCS), thromboxane synthase (TXS), water (H_2_O), hydroxy fatty acid dehydrogenase (HFAD), and soluble epoxy hydrolase (sEH). In panels A and C, OXL levels are displayed as fold-change relative to PRE levels for each treatment group; dots represent individual datapoints; and differences between groups are denoted by **P<0.01, *P<0.05 and tendencies by ^*P*<0.10; n=4. Relative concentrations of AA-derived OXL **(C)** and LA-derived OXL **(D)** released by cultured bovine adipocytes following 3 h of exposure to control media (BAS) or LPS (1 µg/mL). In panels C and D, dots represent individual datapoints; differences between groups are denoted by **P<0.01, *P<0.05, and tendencies by ^*P*<0.10; n=6.

Compared to SAL, LPS increased the 5-LOX AA-derived product 18-carboxy-dinor-LTB_4_ in plasma +2H and +12H (**Figure 4A**). A tendency for the same pattern was observed in 5,6-DiHETE at both time points post-infusion. LPS tended to enhance 5-HETE, a product of 12-LOX, at +12H, but not at +2H (**Figure 4A**). Compared to SAL, products of the 15-LOX pathway, 15-HETE and 15-epi-LXA_4_, exhibited opposing trends in LPS, with 15-HETE levels elevated and 15-epi-LXA_4_ levels reduced at both +2H and +12H (**Figure 4A**). LPS tended to enhance 15-OxoETE concentrations at +12H compared to SAL (**Figure 4A**). LPS increased the COX-derived TXB_2_ 17-fold over SAL at +2H; this gap lessened to 3-fold at +12H (**Figure 4A**). LPS tended to increase the prostanoid-derived 6-keto-PGF1α and the CYP metabolite 11-HETE at +2H and +12H relative to SAL at each respective time point (**Figure 4A**). Notably, compared to SAL, LPS increased adipocytes’ release of 5-HETE and 6-keto-PGF1α into the media (**Figure 4B**).

### 3.5 Plasma linoleic acid-derived oxylipins are elevated following exposure to endotoxin

LPS augmented plasma content of LOX-derived LA metabolites 9- and 13-HODE at +2H compared with SAL (**Figure 4C**). Additionally, LPS increased plasma content of the CYP product 9,10-DiHOME at +2H compared to SAL (**Figure 4C**). LPS tended to increase 9,10-DiHOME at +12H and 12,13-DiHOME at +2H (**Figure 4C**). However, LPS increased plasma 12,13-EpOME content at +12, but not at +2H, compared to SAL (**Figure 4C**). Exposure to LPS increased adipocytes’ release of 13-HODE into the media, corresponding with the metabolite’s changes in plasma (**Figure 4D**). However, in contrast with systemic observations, LPS reduced adipocytes’ release of 9,10-diHOME and 12,13-EpOME compared to CON (**Figure 4D**).

### 3.6 Docosahexaenoic acid-based oxylipin concentrations are altered following endotoxin exposure

**Figure 5:**
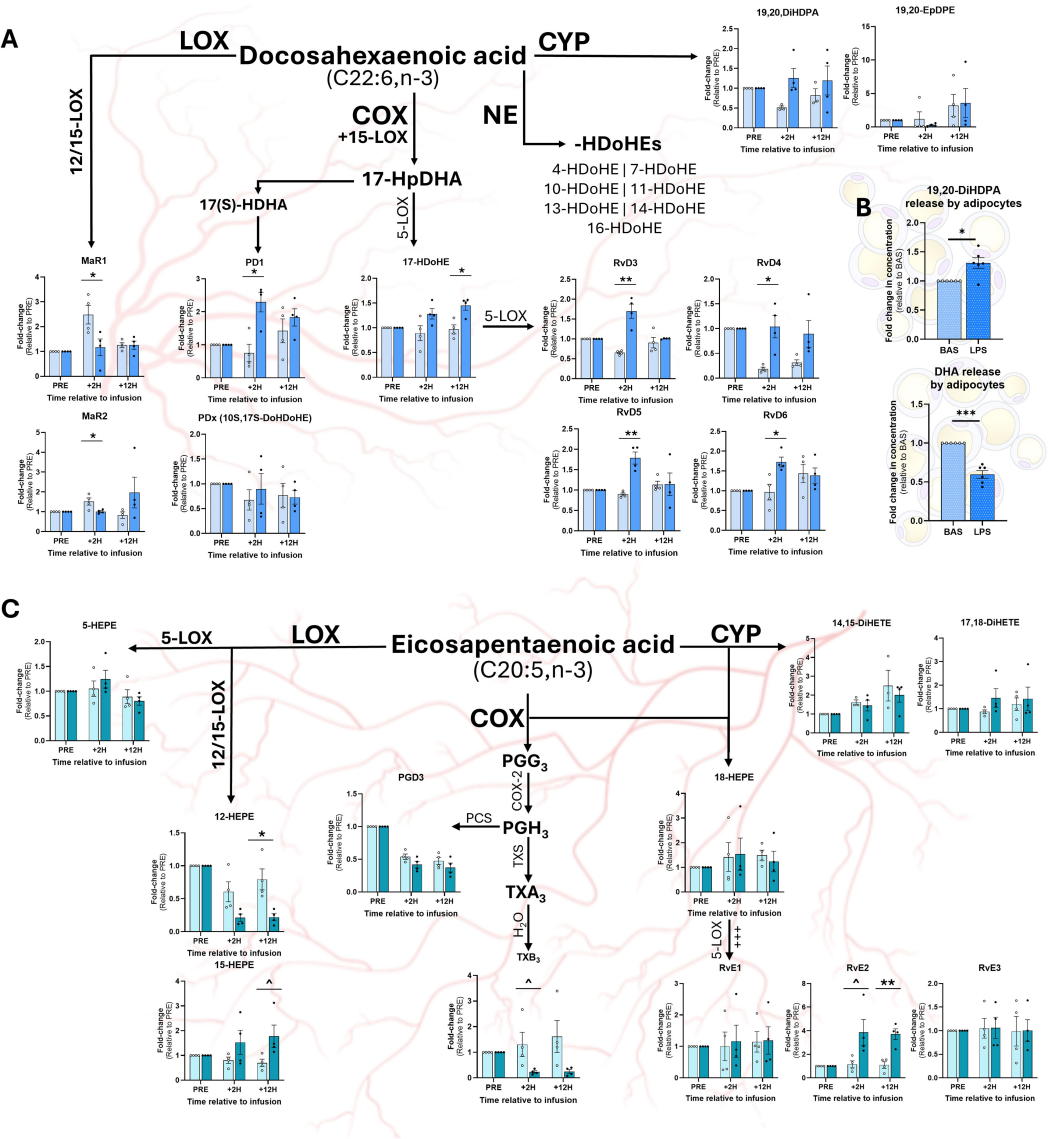
Relative concentrations of omega-3 fatty acid-based oxylipins in plasma and adipocyte culture medium following exposure to LPS. Abundance of docosahexaenoic acid (DHA; C22:6, n-3)-derived OXL in plasma **(A)** and adipocyte culture medium **(B)** and eicosapentaenoic acid (EPA; C20:5)-derived OXL in plasma **(C)**. For plasma OXL quantification, samples were collected prior to (PRE), 2 h following (+2H), and 12 h after (+12H) infusion with saline (SAL; open circles) or bacterial lipopolysaccharide (LPS; closed circles; **(A, C)**. Major enzymatic (lipoxygenase, LOX; cyclooxygenase, COX; cytochrome P450, CYP) and non-enzymatic (NE) synthesis pathways are shown. Oxylipin levels are displayed as fold-change relative to PRE levels for each treatment group. Dots represent individual datapoints. Differences between groups are denoted by **P<0.01, *P<0.05 and tendencies by ^*P*<0.10; n=4. **(B)** Relative concentrations of DHA and DHA-derived OXL released by cultured bovine adipocytes following 7 h of exposure to control media (BAS) or LPS (1 µg/mL). Dots represent datapoints; n=6. Differences between groups are denoted by ***P<0.001, *P<0.05; n=6.

At +2H, MaR1 and MaR2 remained unchanged in LPS, yet decreased in SAL relative to baseline (**Figure 5A**). While no differences were found at +2H, LPS increased plasma 17-HDoHE at +12H (**Figure 5A**). Compared to SAL, LPS increased plasma content of the 15-LOX product protectin D1 (PD1) at +2H, however, no difference was observed between treatments at +12H (**Figure 5A**). Compared to SAL, LPS increased the concentrations of all quantified Rv (RvD3, RvD4, RvD5, and RvD6) at +2H; however, at +12H, there was no effect of treatment (**Figure 5A**). No differences were observed between treatments in non-enzymatically derived plasma -HDoHE (4-, 7-, 10-, 11-, 13-, 14-, or 16-HDoHE) at any time point (data not shown). Contrasting with the changes observed in plasma, LPS attenuated adipocyte release of DHA (**Figure 5B**). However, LPS upregulated adipocytes release of 19,20-DiHDPA compared to CON (**Figure 5B**).

### 3.7 Endotoxin exposure elevates COX-, LOX-, and CYP-derived eicosapentaenoic acid-based oxylipins in plasma

At +12H, LPS reduced plasma content of the 12-LOX EPA-based product, 12-HEPE, but was unchanged at +2H, compared to SAL (**Figure 5C**). Similarly, LPS tended to reduce plasma content of the COX-derived metabolite TXB_3_ at +12H (**Figure 5C**). In contrast, LPS tended to increase plasma content of 15-HEPE, a product of 15-LOX, compared to SAL at +12H (**Figure 5C**). While no differences were observed for RvE1 or RvE3 between treatments, LPS tended to increase resolvin E2 (RvE2) at +2H and augmented RvE2 + 12H (**Figure 5C**) compared to SAL. No differences were observed in adipocyte production of EPA-based OXL upon LPS exposure (data not shown).

### 3.8 Endotoxemia does not alter plasma docosapentaenoic acid-derived oxylipin levels

Although LPS increased plasma DPA compared to SAL, there was no effect of treatment on the DPA-based OXL PD1_n-3,DPA_, RvD5_n-3,DPA_, or AT-RvD5_n-3,DPA_ (data not shown).

### 3.9 Plasma oxylipin concentrations correlate with respiratory and heart rates

**Figure 6:**
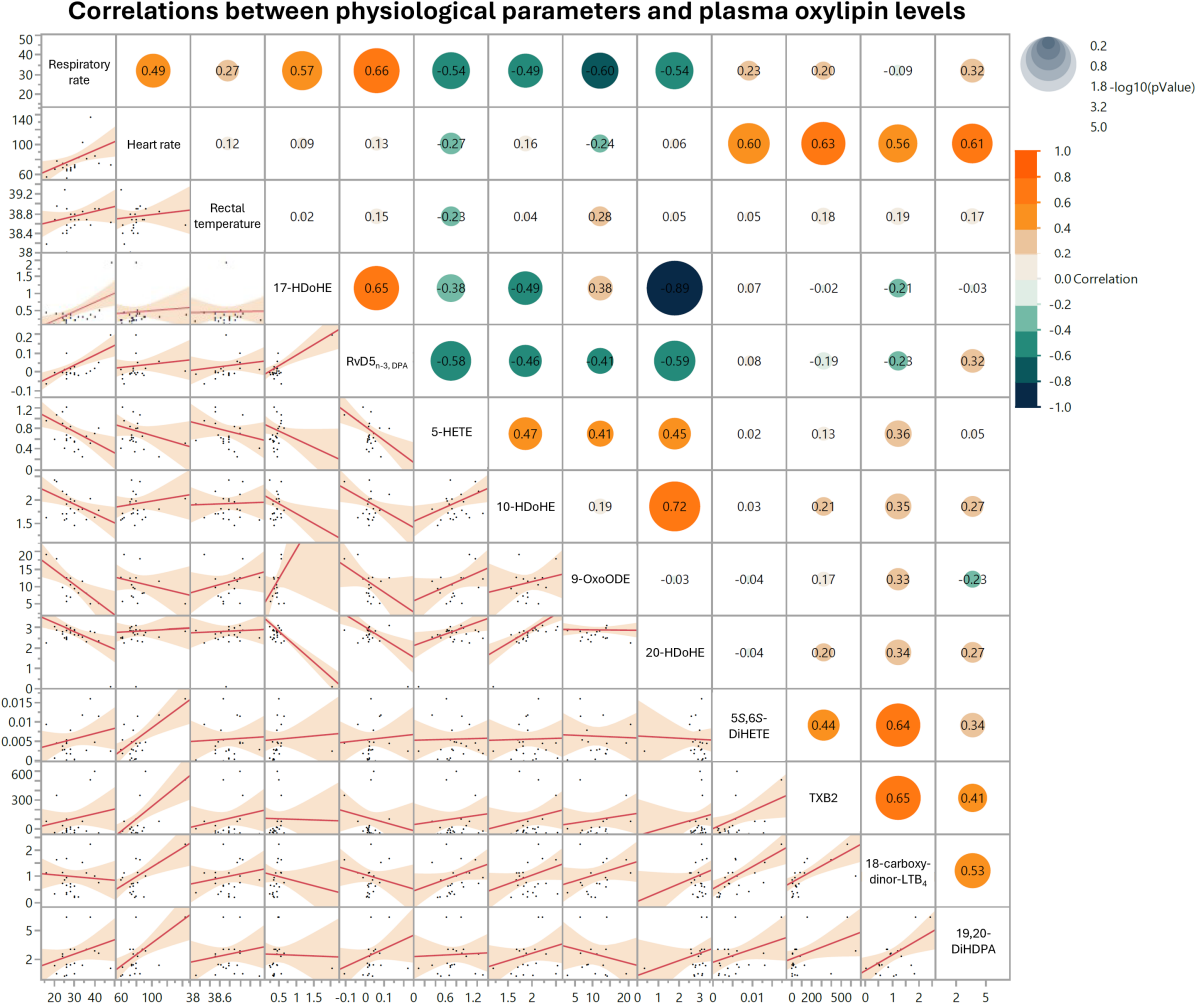
Matrix depicting top 10 significant correlations between respiratory rate, heart rate, rectal temperature, and oxylipin levels in cows treated with lipopolysaccharide (LPS) or sterile saline (SAL). Respiratory rates are shown in breaths per minute, heart rates in beats per minute, and rectal temperatures in degrees Celsius (°C). Oxylipins are shown in ng per mL plasma. In each scatter plot within the matrix’s lower left half, dots represent individual data points across all time periods and both treatment groups. In the upper right half of the matrix, dot sizes correspond to comparison log_10_(*P*-value), dot colors represent correlation strength and directionality, and pairwise correlation values are included in each dot.

Our multivariate analysis revealed a strong positive correlation between respiratory rate and plasma content of RvD5_n-3, DPA_ (**Figure 6**). Respiratory rate and plasma content of 17-HDoHE levels also demonstrated a positive association (**Figure 6**). Negative correlations were observed between respiratory rate and 5-HETE, 10-HDoHE, 9-oxoODE, and 20-HDoHE (**Figure 6**). Positive correlations were identified between heart rate and 5,6-DiHETE, TXB_2_, 18-carboxy-dinor-LTB_4_, and 19,20-DiHDPA (**Figure 6**). No significant correlations were detected between rectal temperature and plasma content of OXL (**Figure 6**).

## 4 Discussion

### 4.1 LPS infusion induces characteristic symptoms of endotoxemia in cows

The clinical responses to LPS infusion observed in cows—tachycardia, fever, tachypnea, salivation, polyuria, increased defecation, and cold extremities—underscore our model’s capacity to reliably recapitulate the acute inflammatory responses observed in humans and other species following single LPS infusions (5). Our results are consistent with previous reports in cows, wherein fevers peaked at 4 h post-infusion and respiratory rates were highest at +2H (40). These well-described symptoms are considered hallmarks of an acute inflammatory response to endotoxemia, reflecting the systemic activation of immune pathways triggered by endotoxin exposure (41). Our findings further validate the use of *in vivo* LPS infusion as a reproducible method to study endotoxemia *in vivo*, providing a robust platform to investigate the downstream biochemical and metabolic changes associated with this condition. Moreover, this model enables further exploration of the role of bioactive lipid mediators, such as OXL, in modulating the inflammatory response and potentially influencing the recovery and health outcomes with translational potential across species.

### 4.2 Oxylipin profiles change in response to endotoxemia

We report, for the first time, that LPS infusion-induced endotoxemia triggers a dynamic and complex systemic OXL response, with distinct lipid mediator pathways activated in a time-dependent manner in dairy cows. The observed alterations in specific OXL pathways, particularly the COX-derived EPA-based and LOX-derived AA-based OXL, indicate that our *in vivo* model of endotoxemia recapitulates the rapid OXL biosynthetic response to endotoxin observed *in vitro* (42). The early and sustained changes in these key mediators may reflect an adaptive or maladaptive immune response to endotoxin, suggesting that OXL play critical roles in the pathogenesis and clinical progression of endotoxemia. Moreover, the persistent alterations in COX-derived EPA-based OXL may indicate a shift toward anti-inflammatory and pro-resolving responses, as COX-synthesized EPA-derived OXL are generally regarded as less inflammatory than their AA-derived counterparts.

In contrast, the sustained increase in LOX-derived AA-based OXL, including LT and HETE, could contribute to a dysfunctional inflammatory response during endotoxemia. Previous research highlights LT and HETE as key mediators of neutrophil chemotaxis, vascular permeability, and immune cell activation (43–45). The persistence of these pathways observed in the present study suggests that the inflammatory cascade and OXL production triggered by LPS does not resolve fully within the observed timeframe, potentially contributing to the chronic aspects of endotoxemia, such as prolonged immune activation, tissue damage, and end-stage organ failure (46, 47).

The distinct separation observed in OXL profiles between the LPS and SAL treatments at +2H underscores the rapid and profound impact of endotoxin exposure on systemic lipid mediator production. The clear distinction in metabolite profiles *in vivo* is in line with a robust inflammatory response initiated early after LPS exposure, as reported previously (48, 49). The trend toward similarity in OXL profiles at +12H suggests that, while the acute effect of inflammation diminishes over time, residual effects of endotoxin persist, particularly through sustained alterations in key OXL synthesizing pathways.

### 4.3 Elevation of n-3 and n-6 fatty acids follow endotoxin exposure

Endotoxin exposure increased plasma concentrations of both n-3 and n-6 FA at +2H, including AA, ALA, DPA, EPA, and LA. The heightened levels of these FA suggest that LPS triggers the hydrolysis and release of FA from cellular stores (e.g., phospholipids and neutral lipids), likely to fuel the synthesis of bioactive lipid mediators as part of the immune response (33). The sustained elevations in plasma AA and sizeable increase in DHA concentrations in both plasma and adipocyte media further support the role of FA in sustained inflammatory signaling and immune modulation during endotoxemia.

### 4.4 Arachidonic acid-based oxylipins are differentially modulated in response to endotoxin

Endotoxin exposure leads to distinct and time-dependent responses in AA-derived OXL, suggesting a complex and dynamic response to systemic inflammation. The upregulation of the 5-LOX byproduct 18-carboxy-dinor-LTB_4_ at both +2H and +12H highlights that stimulation of leukotriene synthesis is prolonged following LPS exposure. This molecule is a metabolite of LTB_4_, a potent pro-inflammatory mediator involved in neutrophil recruitment and activation, which contributes to sustained inflammation during endotoxemia (50). In rodents and humans, the 5-LOX-derived metabolite, 5-HETE, is produced by neutrophils in response to bacterial pathogens and their remnants, including LPS (51). Notably, 5-HETE and its isomers are potent chemoattractants that induce neutrophil migration and macrophage infiltration (52, 53). In the present study, 5-HETE levels tended to be elevated in plasma at +12H, but not +2H, in cows treated with LPS, suggesting this OXL may be involved in the latter stages of the inflammatory response. However, 5-HETE release by adipocytes was dramatically elevated following 3 h of LPS exposure, suggesting that AT may play a more immediate role in 5-HETE production during endotoxemia. Moreover, this finding suggests that adipocytes could significantly contribute to the early inflammatory response, potentially serving as an initial chemoattractant source to prime the infiltration of immune cells into AT commonly observed during inflammatory disease (54, 55).

The trend toward elevated plasma 5,6-DiHETE, a dihydroxy derivative of leukotriene A_4_ (LTA_4_), in response to LPS at +2H and +12H further suggests that activation of the 5-LOX pathway is sustained, reinforcing its potential significance in mounting the immune response to endotoxemia. In fact, several studies have identified anti-inflammatory and pro-resolving characteristics associated with this molecule, including its capacity to reduce vascular permeability and endothelial barrier disruption (56, 57).

Several COX-derived, AA-based OXL were upregulated systemically in response to LPS, including TXB_2_, which was elevated nearly 17-fold at +2H, and 3-fold at +12H. This sharp rise suggests that thromboxane-mediated pro-thrombotic and vasoconstrictive responses occur during the acute phase of endotoxemia. The attenuation of plasma TXB_2_ at +12H suggests that tapering of the thromboxane surge occurs, although residual effects of COX activation persist. Notably, no differences were observed in adipocytes’ TXB_2_ production following LPS exposure, suggesting that the systemic effects observed may occur independently of adipocytes, rather, through production by platelets or immune cells (58).

The COX metabolite 6-keto-PGF1α, a stable product of prostacyclin, tended to be elevated at +2H and was markedly greater at +12H in response to LPS, reflecting sustained vascular and endothelial activation. These findings are in line with the well-established role of prostacyclins in regulating blood flow, limiting platelet aggregation, and inhibiting leukocyte adhesion (59–61). Interestingly, production of 6-keto-PGF1α was drastically elevated in adipocytes following LPS exposure, suggesting that AT may contribute actively to the vascular and endothelial remodeling seen during endotoxin-induced inflammation (62).

The CYP-derived 11-HETE exhibited a pattern in plasma similar to 6-keto-PGF1α, increasing at both +2H and +12H in LPS compared to SAL. As no differences were observed in adipocyte production of 11-HETE in response to LPS exposure, these effects further emphasize that LPS persistent endothelial and vascular effects during systemic inflammation occur independently of adipocyte responses. Moreover, 11-HETE is involved in modulating vascular tone and inflammation, highlighting the multifaceted involvement of CYP pathway in regulating endotoxin-induced vascular dysfunction, particularly in the acute phase of inflammation [as reviewed in (63)].

Also important to consider is that not all AA-derived OXL exhibit pro-inflammatory actions. Of note is the CYP-derived, pro-resolving mediator 15-epi-LXA_4_, which attenuates cytokine release and enhances pathogen clearance in human and murine models of pneumonia (64). We found that LPS treatment decreased plasma content of this compound, a stable and longer-acting derivative of lipoxin A_4_ (65). This finding in our bovine model contrasts with those previously described in the lungs of mice, which report that 15-epi-LXA_4_ increases following LPS exposure (64). These divergent responses between species may reflect differences in the regulation of lipoxin pathways between species, across tissues, or varying roles of 15-epi-LXA_4_ in systemic versus local inflammatory contexts.

### 4.5 Linoleic acid-based oxylipins and their involvement in endotoxemia

Several LA-derived metabolites were altered in plasma following LPS infusion, with elevated levels of the LOX-derived 9-HODE and 13-HODE observed at +2H. These metabolites, which are known markers of oxidative stress and inflammation, may contribute to the tissue damage and cellular dysfunction frequently observed during endotoxemia. Of equal importance to note, however, are the pro-lipogenic, PPARγ-mediated effects exhibited by these HODE, which may serve as a negative feedback loop to limit AT lipolysis during endotoxemia (11).

Additionally, the CYP-derived product, 9,10-DiHOME was elevated in plasma at +2H, indicating upregulated activity of the CYP-to-soluble epoxide hydrolase (sEH) pathway in the early phase of endotoxin-induced inflammation (66). A trend toward higher plasma levels of 9,10-DiHOME at +12H and 12,13-DiHOME at +2H in response to LPS suggests these metabolites may play key roles in modulating the inflammatory response over a more extended period. Moreover, LPS increased plasma 12,13-EpOME at +12H, but not +2H, pointing toward a time-dependent activation of the CYP and sEH pathways that may contribute to the progression of inflammation and vascular permeability in the later stages of endotoxemia. In contrast, both 9,10-DiHOME and 12,13-EpOME production were decreased in adipocytes exposed to LPS, suggesting that endotoxin may suppress local synthesis of these products, perhaps to limit inflammation within AT (14). The discrepancy observed between plasma and adipocyte DiHOME and EpOME levels suggests a compartmentalized regulation of CYP metabolites during endotoxemia, with circulating levels reflecting an upregulation to support systemic inflammatory responses while, within AT, downregulated production of these OXL to limit local inflammation and maintain metabolic homeostasis (67). Collectively, our observations underscore the importance of LA-derived metabolites in the inflammatory cascade, with AT potentially functioning as both a target and source of LA-based OXL during systemic inflammation.

### 4.6 Docosahexaenoic acid-based oxylipins and the resolution of inflammation

DHA-derived OXL, including resolvins (Rv), protectins (PD), and maresins (MaR) are central to the resolution of inflammation. Interestingly, while exposure to LPS increased several D-series Rv (RvD3, RvD4, RvD5, and RvD6) at +2H, no differences were observed at +12H, suggesting a short-lived spike in the abundance of pro-resolving mediators early in the inflammatory response. These findings may highlight an attempt by the immune system to counteract the pro-inflammatory signals generated by AA-derived metabolites. However, the lack of sustained elevations in Rv beyond +2H may indicate that the resolution phase of inflammation is not adequate during endotoxemia, which may contribute to chronic inflammation and delayed recovery times. We speculate that this shift may be attributable to rapid exhaustion of DHA reserves within cell phospholipid membranes or lipid droplets during endotoxemia. This concept aligns with findings in human and murine macrophages, which demonstrate that D-series Rv activate phospholipase D, freeing lipid-containing phosphatidic acids and cholines from cell membranes (68, 69). Important to note, however, is that this response may also be driven by changes in oxidative enzymes’ activation status and preferences for specific FA substrates during inflammatory challenges.

Similarly, the 15-LOX-derived pro-resolving metabolite, PD1, was elevated at +2H, but returned to baseline levels at +12H. In LPS-stimulated macrophages of primates, PD1 exerts its anti-inflammatory effects through stimulation of peroxisome proliferator-activated receptors α and γ (PPARα, PPARγ), which inhibit NF-κB translocation and downregulate transcription of pro-inflammatory genes encoding TNF-α, IL-6, and iNOS (16, 70). Taken together, these findings suggest that, while pro-resolving mediators are initially upregulated during LPS-mediated inflammation, their effects may be transient and limited in the context of chronic endotoxin exposure. Moreover, the transient spike observed in PD1 levels implies that anti-inflammatory pathways are activated but may be insufficiently sustained to fully outweigh the pro-inflammatory cascades initiated by endotoxemia.

While the LPS treatment did not increase plasma 19,20-DiHDPA, its production was notably enhanced in adipocytes following endotoxin exposure. This finding suggests that AT may selectively increase the synthesis of 19,20-DiHDPA as part of the localized response to pathogens, independent of systemic changes detectable in plasma. Of interest, 19,20-DiHDPA is associated with anti-inflammatory effects and may act to offset the production of pro-inflammatory mediators within AT. The upregulation of this metabolite’s synthesis in adipocytes, specifically, suggests a potential protective mechanism within AT aimed at mitigating the negative consequences of prolonged inflammation and preserving adipocyte functionality during endotoxemia.

### 4.7 Endotoxemia modulates eicosapentaenoic acid-based oxylipin levels

The differential regulation of EPA-derived OXL during endotoxin exposure reflects a complex interplay between pro-inflammatory and pro-resolving pathways. The suppression of specific LOX and COX metabolites, such as 12-HEPE and TXB_3_, suggests a potential shift away from certain inflammatory mediators in response to endotoxemia. This reduction could indicate an adaptive response aimed at limiting excessive inflammation in the latter stages of endotoxin exposure, however, this response may impair the immune system’s capacity to effectively clear pathogens.

Conversely, the elevation of 15-LOX products and specific E-series Rvs, such as RvE2, points toward a heightened activation of pathways involved in inflammatory resolution. In fact, RvE2 serves as a competitive antagonist of the AA-derived LTB_4_ and inhibits the activation of its receptor, LTB_4_R1, limiting leukocyte recruitment and activation (71). The increases in pro-resolving mediators observed herein underscore the body’s efforts to counteract pro-inflammatory pathways triggered by endotoxemia, especially as the immune response progresses over time.

### 4.8 Plasma oxylipin levels correspond with changes in physiological parameters

The correlations observed between respiratory and heart rates with specific OXL may suggest that these potent lipid mediators actively influence key symptoms associated with endotoxemia. The strong positive correlation between respiratory rate RvD5_n-3,DPA_—an OXL known for its anti-inflammatory, pro-resolving actions—implies that elevated respiratory rates may be driven by upregulation of resolving pathways in response to inflammatory stimuli. RvD5_n-3,DPA_ has been shown to enhance macrophage-mediated pathogen clearance, which may reflect an adaptive attempt to restore homeostasis in the microenvironments of various organs and limit tissue damage driven by endotoxemia (72, 73). Likewise, the positive association with the DHA-derived RvD- and PD- precursor, 17-HDoHE, further supports OXL roles in respiratory modulation, as prior studies in mice suggest these compounds reduce pulmonary inflammation and promote pathogen clearance by neutrophils and macrophages (74).

Conversely, the negative relationship between respiratory rate and pro-inflammatory 5-HETE suggests that this molecule may intensify inflammatory processes that challenge respiratory function. Past research demonstrates that 5-HETE stimulates constriction of the vasculature and accumulation of fluid in the lungs, which may lead to respiratory distress (75). Moreover, this molecule is known to slow contraction of smooth muscle in pulmonary bronchioles, which may limit gas exchange and blood oxygenation during endotoxemia (76).

Heart rate demonstrated moderate positive correlations with OXL such as TXB_2_, 18-carboxy-dinor-LTB_4_, and 5,6-DiHETE, suggesting that these pro-inflammatory mediators may drive tachycardia and vascular responses commonly seen in endotoxemia. TXB_2_, a stable metabolite of thromboxane A2, induces vasoconstriction and platelet aggregation, potentially increasing heart rate and cardiac workload as part of an acute vascular response to inflammation (77). Additionally, LTB_4_ and its derivatives, including 18-carboxy-dinor-LTB_4_, have been linked to heightened leukocyte activation and endothelial interactions that could intensify systemic inflammation, subsequently increasing heart rate to meet metabolic demands (78). Taken together, these findings indicate that specific OXL may actively drive respiratory and cardiovascular adaptations during endotoxemia, aligning with their known roles in modulating inflammation and vascular responses.

### 4.9 Additional considerations

Our study is not without limitations. While we quantified temporal changes in OXL profiles and their magnitudes in plasma, their tissues of origin remain unknown. Additionally, the transcriptional, translational, and activation statuses of oxidizing enzymes which drive OXL production were not directly quantified in this study. Moreover, the decision to analyze samples at +2H and +12H may have limited our ability to detect changes in plasma OXL with shorter half-lives or delayed response times. While previous evidence by our group demonstrates that 3 h of LPS exposure alters adipocytes’ synthesis of lipid-based mediators and transcription of key oxidizing enzymes (e.g., COX), this model cannot replicate chronic inflammation *in vivo* (33). In addition, previous research indicates that mitochondrial degradation of OXL is upregulated during LPS-induced inflammation, framing the need for additional *in vivo* studies to fully elucidate the dynamic balance between synthesis and degradation of these lipid mediators (79). Although this study has a relatively small sample size, we observed consistent patterns across individuals, and the use of robust statistical methods supports the reliability of our findings. Additional studies with larger cohorts are needed to elucidate mechanistic details about specific OXL pathways. Importantly, as used in the present study, single-infusion models may not capture the full effects of repeat or chronic exposure to LPS on the OXL profile, however, the initial evidence provided herein supports the implementation of a multiple LPS infusion model in cows during negative energy balance for use in future studies.

### 4.10 Conclusions

Our findings demonstrate that LPS infusions induce distinct clinical, physiological, and biochemical responses in dairy cows, recapitulating hallmark inflammatory symptoms of endotoxemia, thereby providing a robust model for studying systemic inflammation. Our results show that dynamic alterations occur in response to endotoxemia, with time-dependent shifts in key FA-derived mediators that suggest they play distinct roles in the initiation, progression, and resolution of inflammation. The observed differential modulation of COX-, LOX-, and CYP-derived metabolites from AA, EPA, DHA, and LA underscores the complex, time-dependent nature of OXL pathways in both promoting and resolving inflammation. While pro-inflammatory AA-derived metabolites persisted throughout our study’s timeframe, the spikes observed in DHA- and EPA-derived pro-resolving mediators suggest an incomplete and, perhaps, impaired resolution of inflammation. Additionally, our results underscore the potential role of AT in OXL production during endotoxemia, with adipocytes serving as both a source and modulator of inflammatory mediators. The distinct responses observed between *in vivo* plasma and *in vitro* adipocyte models point to a potential protective mechanism within AT that may buffer local inflammation while supporting systemic responses necessary for pathogen clearance. Altogether, these insights into the OXL response to LPS provide valuable directions for future therapeutic strategies aimed at targeting lipid mediator pathways to mitigate systemic inflammation and improve outcomes in human and veterinary patients challenged with endotoxemia.

## Data Availability

The data generated and used in the analysis of results reported in this manuscript are available on Figshare at the following URL: https://doi.org/10.6084/m9.figshare.28914443.
