# Supplementary material for: Dynamics of oxylipin biosynthesis in systemic inflammation: insights from a large animal model of endotoxemia
Source: Front Immunol. 2025 Jun 16;16:1595888. doi: 10.3389/fimmu.2025.1595888 (PMC12206649; doi:10.3389/fimmu.2025.1595888)
Supplement: Supplementary file 1 [file Table1.docx]

| **Component Name** | **IS Name** | **Expected RT (min)** | **Precursor (m/z)** | **Fragment (m/z)** |
| --- | --- | --- | --- | --- |
| PGE1-D4 | N/A | 6.69 | 357.2 | 277.1 |
| RvD2-D5 | N/A | 6.92 | 380.3 | 175.1 |
| LTB4-D4 | N/A | 10.22 | 339.2 | 197.2 |
| 15-HETE-D8 | N/A | 12.58 | 327.2 | 226.1 |
| 14,15-EpETrE-D11 | N/A | 13.34 | 330.3 | 219.1 |
| PGE2 | PGE1-D4 | 6.43 | 351.2 | 271.1 |
| PGE3 | PGE1-D4 | 2.19 | 349.2 | 269.1 |
| PGD2 | PGE1-D4 | 6.85 | 351.2 | 271.1 |
| PGD3 | PGE1-D4 | 6.22 | 349.2 | 269.1 |
| PGF2a | PGE1-D4 | 7.25 | 353.2 | 193.1 |
| PGF3a | PGE1-D4 | 6.1 | 351.2 | 193.1 |
| 6-keto PGF1α | PGE1-D4 | 4.74 | 369.2 | 163.1 |
| TXB2 | PGE1-D4 | 6.35 | 369.2 | 195.1 |
| TXB3 | PGE1-D4 | 5.2 | 367.2 | 195.1 |
| LTB4 | LTB4-D4 | 10.41 | 335.2 | 195.1 |
| 20-hydroxy LTB4 | LTB4-D4 | 5.21 | 351.2 | 195.1 |
| 20-COOH LTB4 | LTB4-D4 | 2.75 | 365.2 | 195.1 |
| 18-carboxy dinor LTB4 | LTB4-D4 | 2.4 | 337.2 | 195.1 |
| LTB5 | LTB4-D4 | 9.7 | 333.2 | 195.1 |
| 5(S),6(S)-DiHETE | LTB4-D4 | 12.5 | 335.2 | 163.1 |
| 5(S),12(S)-DiHETE | LTB4-D4 | 11.67 | 335.2 | 195.2 |
| 5(S),15(S)-DiHETE | LTB4-D4 | 10.05 | 335.2 | 201.1 |
| 8(S),15(S)-DiHETE | LTB4-D4 | 9.45 | 335.2 | 235.1 |
| 5(S),15(S)-DiHEPE | LTB4-D4 | 10.3 | 333.2 | 253.2 |
| 9-HODE | 15-HETE-D8 | 13 | 295.2 | 171.1 |
| 13-HODE | 15-HETE-D8 | 12.55 | 295.2 | 195.1 |
| 5-HETE | 15-HETE-D8 | 14.44 | 319.2 | 115.1 |
| 11-HETE | 15-HETE-D8 | 13.25 | 319.2 | 167.1 |
| 12-HETE | 15-HETE-D8 | 13.53 | 319.2 | 179.1 |
| 15-HETE | 15-HETE-D8 | 13.06 | 319.2 | 219.2 |
| 20-HETE | 15-HETE-D8 | 12.27 | 319.2 | 257.1 |
| 5-HEPE | 15-HETE-D8 | 13 | 317.2 | 115.1 |
| 12-HEPE | 15-HETE-D8 | 12.2 | 317.2 | 179.1 |
| 15-HEPE | 15-HETE-D8 | 13.1 | 317.2 | 219.1 |
| 18-HEPE | 15-HETE-D8 | 11.67 | 317.2 | 259.1 |
| 4-HDoHE | 15-HETE-D8 | 14.8 | 343.2 | 101.1 |
| 7-HDoHE | 15-HETE-D8 | 13.91 | 343.2 | 141.1 |
| 8-HDoHE | 15-HETE-D8 | 14.03 | 343.2 | 189.1 |
| 10-HDoHE | 15-HETE-D8 | 13.51 | 343.2 | 153.1 |
| 11-HDoHE | 15-HETE-D8 | 13.65 | 343.2 | 121.1 |
| 13-HDoHE | 15-HETE-D8 | 13.24 | 343.2 | 193.1 |
| 14-HDoHE | 15-HETE-D8 | 13.34 | 343.2 | 161.1 |
| 16-HDoHE | 15-HETE-D8 | 13 | 343.2 | 233.1 |
| 17-HDoHE | 15-HETE-D8 | 13.08 | 343.2 | 201.1 |
| 20-HDoHE | 15-HETE-D8 | 12.78 | 343.2 | 241.1 |
| 9(10)-EpOME | 14,15-EpETrE-D11 | 13.76 | 295.2 | 171.1 |
| 12(13)-EpOME | 14,15-EpETrE-D11 | 13.59 | 295.2 | 195.1 |
| 9,10-DiHOME | LTB4-D4 | 10.72 | 313.2 | 201.1 |
| 12,13-DiHOME | LTB4-D4 | 10.34 | 313.2 | 183.1 |
| 5,6-DiHETE(EPA) | LTB4-D4 | 11.1 | 335.2 | 145.1 |
| 9-OxoODE | 15-HETE-D8 | 12.45 | 293.2 | 185.1 |
| 13-OxoODE | 15-HETE-D8 | 12.09 | 293.2 | 113.1 |
| LXA4 | LTB4-D4 | 8.12 | 351.2 | 217.1 |
| 15-epi LXA4 | LTB4-D4 | 9.4 | 351.3 | 217.2 |
| 15-oxo LXA4 | LTB4-D4 | 8.3 | 349.2 | 189 |
| LXA5 | LTB4-D4 | 6.94 | 349.2 | 215.1 |
| LXB4 | LTB4-D4 | 8.01 | 351.2 | 221.1 |
| RvD1 & AT-RvD1 | RvD2-D5 | 8.05 | 375.2 | 141.1 |
| RvD2 | RvD2-D5 | 7.4 | 375.2 | 175.1 |
| RvD3 | RvD2-D5 | 7.6 | 375.3 | 147.1 |
| AT-RvD3 | RvD2-D5 | 7.1 | 375.2 | 147.2 |
| RvD4 | RvD2-D5 | 9.39 | 375.3 | 131.1 |
| AT-RvD4 | RvD2-D5 | 9.3 | 375.2 | 131.1 |
| 22-OH-PD1 | RvD2-D5 | 6.1 | 375.2 | 204.2 |
| RvE1 | RvD2-D5 | 4.9 | 349.2 | 195.1 |
| RvD5 | LTB4-D4 | 10.22 | 359.2 | 199.1 |
| RvD6 (4,17-DiHDoHE) | LTB4-D4 | 10.5 | 359.2 | 159.1 |
| AT-RvD6 (4_17-DiHDoHE) | LTB4-D4 | 10.9 | 359.2 | 215.1 |
| RvD5(n-3,DPA) (7,17-DiHDoPE) | LTB4-D4 | 10.3 | 361.2 | 219.1 |
| AT-RvD5(n-3_DPA) (7,17-DiHDoPE) | LTB4-D4 | 10.4 | 361.2 | 201.1 |
| RvE2 | LTB4-D4 | 9.1 | 333.2 | 213.1 |
| RvE3 | LTB4-D4 | 10.7 | 333.2 | 201.1 |
| PD1 | LTB4-D4 | 11.16 | 359.2 | 206.1 |
| AT-PD1 | LTB4-D4 | 10.1 | 359.2 | 206.2 |
| PDx (10S,17S-DiHDoHE) | LTB4-D4 | 9.7 | 359.2 | 153.1 |
| PD1(n-3,DPA) | LTB4-D4 | 10.3 | 361.2 | 155.1 |
| Maresin 1 | LTB4-D4 | 10.23 | 359.2 | 177.1 |
| Maresin 2 | LTB4-D4 | 10.63 | 359.2 | 221.2 |
| Maresin 1(n-3,DPA) | LTB4-D4 | 10.6 | 361.2 | 223.1 |
